# *Ziziphus mauritiana* Leaves Normalize Hormonal Profile and Total Cholesterol in Polycystic Ovarian Syndrome Rats

**DOI:** 10.3390/plants12142599

**Published:** 2023-07-09

**Authors:** Thippeswamy Boreddy Shivanandappa, Maheswari Chinnadhurai, Geetha Kandasamy, Rajalakshimi Vasudevan, Gigi Sam, Anjana Karunakarannair

**Affiliations:** 1 Department of Biomedical Sciences, College of Pharmacy, Shaqra University, Al-Dawadmi Campus, Al-Dawadmi 11961, Saudi Arabia; drswamy@su.edu.sa (T.B.S.); anju@su.edu.sa (A.K.); 2 Department of Pharmacy Practice, College of Pharmacy, Shaqra University, Al-Dawadmi Campus, Al-Dawadmi 11961, Saudi Arabia; 3 Department of Clinical Pharmacy, College of Pharmacy, King Khalid University, Abha 61421, Saudi Arabia; glakshmi@kku.edu.sa; 4 Department of Pharmacology, College of Pharmacy, King Khalid University, Abha 61421, Saudi Arabia; raja@kku.edu.sa; 5 Department of Pharmaceutical Sciences, College of Pharmacy, Shaqra University, Al-Dawadmi Campus, Al-Dawadmi 11961, Saudi Arabia; gigisam@su.edu.sa

**Keywords:** Indian Jujube, PCOS, letrozole, clomiphene citrate

## Abstract

In the present study, the beneficial effect of leaves of *Ziziphus mauritiana* on testosterone, estradiol, progesterone, LH hormones, blood glucose, and total cholesterol levels in the experimentally induced polycystic ovaries of female Sprague Dawley rats were evaluated. Letrozole was used to induce PCOS in rats, and clomiphene citrate was used as a standard control. This study was carried out in vivo on 30 female rats where group I received normal saline and group II to V were treated with letrozole (1 mg/kg/day), which was dissolved in normal saline orally for 21 days to induce PCOS. After PCOS induction, test groups III and IV were orally treated with ZMME at a dose of 100 mg/kg and 200 mg/kg for 14 days, respectively, and group V was treated with clomiphene citrate (2 mg/kg) orally for 14 days. At the end of the experimental period, the animals were sacrificed by cervical dislocation, and blood samples were collected by cardiac puncture. After blood collection, the ovaries were removed and weighed. The results showed that *Ziziphus mauritiana* normalized all hormones and total cholesterol levels. The HPTLC profile showed the presence of gallic acid, rutin, quercetin, and ursolic acid. Many studies have reported that quercetin is effective against PCOS and its complications; it suppresses insulin resistance and reduces testosterone and LH levels. The present study showed an improvement in the inflammatory microenvironment of the ovarian tissue in the PCOS rat model. This research concluded that the leaves of *Ziziphus mauritiana* have potential efficacy in the treatment of PCOS by normalizing abnormal hormones and total cholesterol levels, which could be due to the presence of quercetin in the leaves.

## 1. Introduction

Infertility is a global problem affecting people all over the world; its causes and importance may vary depending on geographic location and socio-economic conditions. According to statistics, every year, 60–80 million couples in the world suffer from infertility [1]. As defined by the International Committee for Monitoring Assisted Reproductive Technology (ICMART) and the World Health Organization (WHO), “Infertility” is a disease of the reproductive system that is characterized by failure to achieve a clinical pregnancy after 12 months or more of regular unprotected sexual intercourse. The most common causes of female infertility are problems with the fallopian tubes and uterus, disorders of the menstrual cycle, sexual disorders, and ovarian failure. Most cases of infertility in women result from problems with producing eggs due to polycystic ovarian syndrome (PCOS); the ovaries may not release an egg regularly, or they may not release a healthy egg. The actual prevalence of PCOS worldwide is unknown because different sets of diagnostic criteria make the comparison of studies difficult, and also data obtained from many geographical regions are limited. However, estimates using the Rotterdam criteria indicate that 15–20% of women are affected with PCOS [2].

PCOS is one of the most common endocrine disorders in women of reproductive age. It is characterized by a combination of hyperandrogenism, chronic oligo/anovulation, and polycystic ovaries, leading to symptoms of infertility, irregular menstruation, acne, and hirsutism. Obesity and insulin resistance are the most common contributing factors to PCOS. Raised insulin levels can cause the ovaries to produce high levels of testosterone, which can impair the normal ovulation process. Treatment options for infertility due to polycystic ovarian disease include lifestyle modifications such as exercise and a low carbohydrate diet to reduce insulin levels and weight loss. Abiraterone acetate normalizes androgen excess in women with a classic 21-hydroxylase deficiency; however, the 17-hydroxylase inhibitory action of this drug blocks cortisol synthesis and causes an ACTH-driven mineralocorticoid excess in patients with intact 21-hydroxylase activity [3]. In women with PCOS, the use of an abiraterone acetate would require additional glucocorticoid supplementation, secure contraception, and, possibly, estrogen therapy to avoid hypoestrogenism. Therefore, it does not appear to be a suitable pharmacologic therapy for most patients with functional androgen excess. Other treatment options for PCOS patients include clomifene citrate as a first-line therapy that can induce ovulation. The second line of treatment is laparoscopic ovarian drilling (LOD): a surgical procedure that can restore ovulation. In vitro fertilization (IVF) is another option where both first-line and second-line therapy are unsuccessful. Women with PCOS undergoing IVF are at significant risk of ovarian hyperstimulation syndrome and are also at increased risk of developing gestational diabetes, pregnancy-induced hypertension, and pre-eclampsia. The success rate of currently available treatment options for PCOS is lower due to side effects and is ineffective in many cases. Due to the above risk factors, in addition to exercise and a lower carbohydrate diet, many PCOS patients take herbal supplements due to their lower side effects and greater efficacy. Only a limited number of herbal remedies are currently available for the treatment of PCOS and have not been evidenced clinically.

*Ziziphus mauritiana* (ZM) belongs to the family Rhamnaceae and is commonly known as Indian jujube or ber; it grows in tropical and sub-tropical regions of the world, including India, Saudi Arabia, Egypt, Japan, Malaysia, Pakistan, Nepal, and Philippines, Southern Africa, Kenya, and Afghanistan. The leaves are used in the treatment of diarrhea, diabetes mellitus, gastric disorder, fever, and pulmonary disorders [4,5]. The leaves are also reported to have wound healing, hepatoprotective, antibacterial, antioxidant, anti-inflammatory, anti-ulcer, and skin rejuvenation activities [6,7,8,9,10,11]. The leaves have been reported for the presence of alkaloids, sapogenin, flavonoids, triterpenoids, and phenolic compounds [12]. An endogenous prostaglandin 12 (PGI2) inducer that was isolated from the leaves of ZM was identified as 1, a novel neo-lignan and a potent vasodilator made in the vascular walls [13]. Although no reports are available on the PCOS activity of ZM in a review of the literature, herbal substances are becoming increasingly common and are replacing known drugs for the treatment of PCOS symptoms. Given our interest in the potential application of the *Ziziphus mauritiana* extract as an alternative medicine and as a treatment for PCOS, our research aimed to evaluate the beneficial effect of the *Ziziphus mauritiana* extract on letrozole-induced polycystic ovarian disease.

## 2. Results

### 2.1. Phytochemical Screening

The methanol extract of leaves of *Ziziphus mauritiana* showed the presence of flavonoids, glycosides, terpenoids, phenols, carbohydrates, proteins, and saponins.

### 2.2. HPTLC Profile

**Figure 1:**
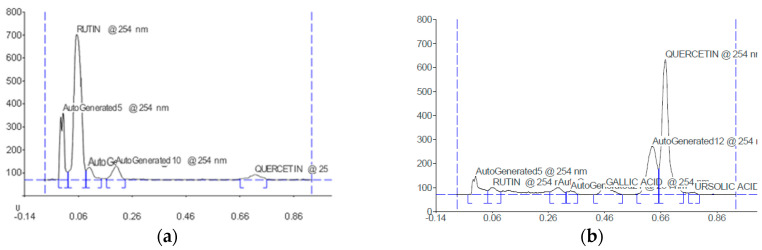

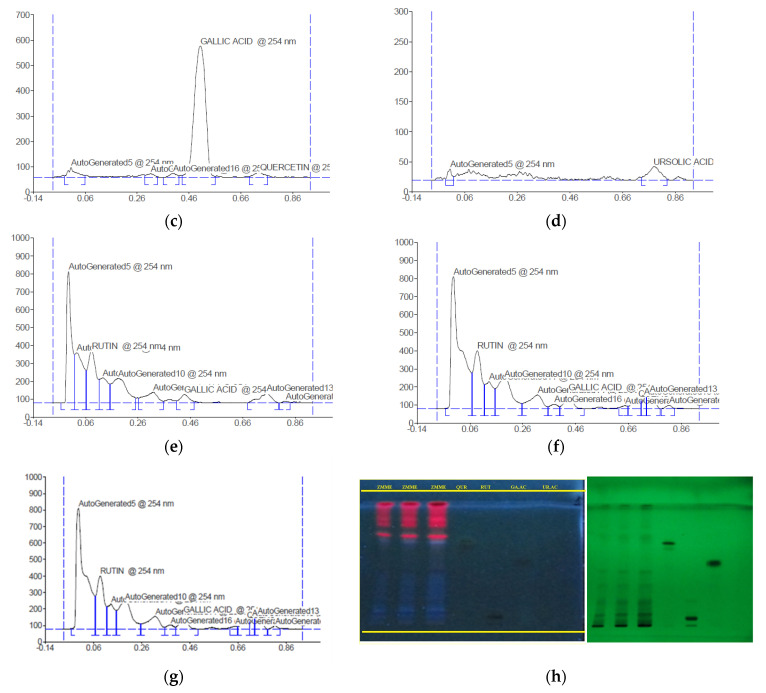
Chromatograms of phytoconstituents scanned at λ_max_ = 254 nm (**a**) rutin (R_f_ = 0.05), (**b**) quercetin (R_f_ = 0.68), (**c**) gallic acid (R_f_ = 0.50), (**d**) ursolic acid (R_f_ = 0.78) and (**e**–**g**) ZM extract (ZMME) at 254 nm (**h**) Chromatograms of ZMME and standards visualized under UV light of wavelength 254 nm. QUR (quercetin), RUT (rutin), GA.AC (gallic acid), UR.AC (ursolic acid).

The chromatograms in Figure 1 specify that all the sample constituents were distinctly segregated without any tailing effect. The results from the HPTLC fingerprint scanned at wavelength 254 nm showed that there were 18 phytoconstituents. The concentration of the phytoconstituents gallic acid, rutin, quercetin, and ursolic acid were found to be 85.77% (R_f_ value = 0.50), 59.54% (R_f_ value = 0.05), 58.27% (R_f_ value = 0.68) and 54.96% (R_f_ value = 0.78), respectively (Table 1, Figure 1a–f)

### 2.3. Acute Toxicity Studies

In an acute toxicity study, the methanolic extract of ZMME was found to be devoid of the mortality of animals and at a dose of 2000 mg/kg body weight. All the animals were found to be normal, and there were no gross behavioral changes until the end of the observation period. This observation revealed that ZMME did not show any signs and symptoms of toxicity and mortality up to 2000 mg/kg dose and was found to be very safe as a maximum tolerated dose (MTD) per the OECD guidelines 423. Hence, 1/20th (100 mg/kg) and 1/10th (200 mg/kg) doses of *Ziziphus mauritiana* extracts were selected for further pharmacological screening.

### 2.4. Effect of Different Drug Treatments on Oestrus Cycle

All rats had regular oestrus cyclicity in the control group. PCOS-induced rats had an irregular oestrus cycle. Treatment with ZMME 100 mg/kg (69.72%), ZMME 200 mg/kg (73.31%), and Clomiphene citrate (75.12%) increased the percentage of rats with regular oestrus cyclicity compared with PCOS rats.

### 2.5. Effect of Drug Treatments on Body Weight and Ovarian Weight

Body weight was significantly (*p* < 0.01) increased in PCOS rats compared to the normal control rats. However, body weight was significantly (*p* < 0.01) decreased in animals treated with ZMME 100, 200 mg/kg, and clomiphene citrate compared to group II. In the PCOS control group, ovarian weight was increased compared to normal control rats. whereas a significant reduction in ovarian weight was observed in ZMME 100, 200 mg/kg and clomifene citrate treated rats (*p* < 0.01) compared to PCOS rats (Table 2).

### 2.6. Effect of Different Treatments on Plasma Progesterone, LH, Estradiol and Testosterone Levels

After the induction of PCOS, testosterone, and LH levels were significantly (*p* < 0.01) increased, while progesterone and estradiol concentrations were decreased (*p* < 0.01) compared to the normal control group. Treatment with ZMME 100, 200 mg/kg (*p* < 0.01), and clomiphene citrate (*p* < 0.01) showed a significant decrease in testosterone and LH levels and a significant increase in the estradiol and progesterone (*p* < 0.01) level compared to PCOS control rats (Table 3).

### 2.7. Effect of Different Treatments on Total Cholesterol and Blood Sugar

PCOS rats had high total cholesterol levels compared to the normal control rats. ZMME 100, 200 mg/kg, and standard groups reduced the serum total cholesterol. A significant improvement in the total cholesterol was observed for ZMME 100, 200 mg/kg, and clomiphene citrate groups and no significant changes were observed for blood sugar in all the treatment groups (Table 4 and Table 5).

### 2.8. Ovarian Histopathology

**Figure 2:**
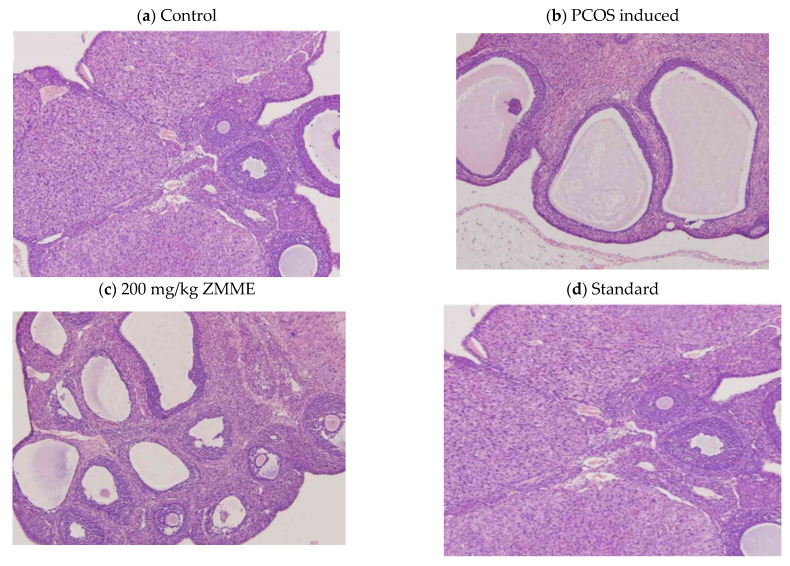
Histopathology. (**a**) Ovary section from control group showing healthy follicles. (**b**) Ovary section from Letrozole-induced PCOS groups showing numerous dilated follicular cysts. (**c**) Section of ovary from ZMME treated (200 mg/kg) group showing a reduced follicular cyst and developed antral follicles. (**d**) Ovary section from standard group showing healthy follicles and less of the follicular cyst.

Ovarian sections from the control rats showed healthy follicles (Figure 2a). Letrozole-treated rats exhibited numerous dilated follicular cysts and thickened the antral follicle theca layer and anovulation (corpora lutea were completely absent) (Figure 2b). Sections from a low dose of ZMME (100 mg/kg) showed no improved histological features and exhibited dilated follicles and a few corpora lutea. The absence of cysts and the presence of healthy normal-sized follicles with a high dose of ZMME (200 mg/kg) were observed. Many corpora lutea and antral follicles were also observed at a high dose (Figure 2c). Clomiphene citrate-treated sections showed the disappearance of cysts and the appearance of healthy follicles (Figure 2d).

## 3. Discussion

Currently, no effective drug therapies are available to treat both the metabolic and reproductive complications of PCOS. Available pharmacotherapies are not effective for PCOS woman with infertility due to side effects. Oral contraceptives are an effective option for the management of PCOS symptoms by decreasing ovarian androgen production, increasing the sex hormone-binding globulin, which limits free testosterone, and regulating menstruation. The potential side effects of oral contraceptives are weight gain which aggravates PCOS, and cardiovascular risks such as hypertension, heart attack, and stroke [14]. Oral contraceptives are not suitable for PCOS woman who plan to conceive. Anti-androgens like spironolactone block androgen receptors, thereby inhibiting the effects of androgens, but produce potential teratogenic effects on the fetus, including hepatotoxicity and hypotension. Insulin-sensitizing agents such as metformin reduce serum androgen and gonadotropins and reduce hyperinsulinemia. Thiazolidinediones improve insulin resistance and hyperandrogenemia [15]. However, Metformin may cause gastrointestinal discomfort, and thiazolidinediones may produce weight gain, which can worsen PCOS. Statins are cholesterol-lowering drugs that inhibit the production of androgens and inhibit the proliferation of ovarian theca cells but can cause hyperglycemia. A selective estrogen receptor modulator like clomiphene citrate binds to estrogen receptors to inhibit gonadotropin release and induce ovulation, causing ovarian hyperstimulation syndrome [16]. Considering the above problems, in the present study, we evaluated methanol extracts of *Ziziphus mauritiana* leaves for PCOS treatment. Letrozole was used to induce PCOS in rats, and it was a third-generation aromatase inhibitor that blocked the conversion of testosterone to estrogen, thus inducing hyperandrogenism [17]. Clomiphene citrate was used as the standard drug, which is most commonly used to induce ovulation in PCOS women [18].

The control group showed a regular oestrus cycle, but the PCOS-treated group showed no regular estrus cycles and was in the diestrus phase. Treatment with ZMME 100, 200 mg/kg, and clomiphene citrate reversed the disestrus phase to the oestrus phase. Ovarian weight and body weight were increased in the PCOS-induced group; however, both body weight and ovarian weight were decreased in ZMME 100, 200 mg/kg, and clomiphene citrate groups. Compared to other test groups, ZMME 200 mg/kg effectively reduced both body weight and ovarian weight, which was comparable with the standard group.

Polycystic ovarian disease is one of the most common causes of female infertility, and the symptoms of PCOS include irregular menstrual cycles, weight gain, and infertility. Physicians diagnose PCOS in women who have had at least three of the following symptoms, including, high androgen levels, a lack of ovulation, and multiple cysts in the ovaries [19,20].

Ovaries produce progesterone and estrogen, which regulates the menstrual cycle. The lack of ovulation in PCOS patients produces abnormal progesterone and estrogen levels. In the present study, progesterone and estradiol levels were lower in the PCOS group, whereas ZMME 100 and 200 mg/kg clomiphene citrate treatment brought back normal progesterone and estrogen levels. The ovaries also produce lower amounts of male hormones called androgens; a higher androgen level in PCOS women deregulates the menstrual cycle, and so women receive fewer periods than normal [21]. In the present study, the testosterone level was elevated in the PCOS-treated group, and the raised level was potentially normalized by a high dose, of ZMME and standard groups. A marginal effect was observed with a low dose of ZMME whereas significant activity was observed for the high dose of ZMME, and this activity was equal to the clomiphene citrate-treated group.

Ovulation is controlled by FSH and LH hormones, where FSH stimulates the ovary to produce a follicle, a sac that contains an egg, and thereafter LH triggers the ovary to release the matured egg [22]. Upon the evaluation of the LH level in the present study, PCOS rats showed increased LH levels, and this was decreased with 100, 200 mg/kg of ZMME and in the standard groups. A total of 200 mg/kg of ZMME effectively reversed the LH level to normal, which was equal to clomiphene citrate. A total of 200 mg/kg of ZMME effectively brought back abnormal hormone levels to normal, which was equal to the clomiphene citrate group. This indicates the potential activity of 200 mg/kg of the methanolic extract of ZM leaves at a higher dose and comparatively less activity at a lower dose.

Dyslipidaemia in PCOS is prevalent and characterized by elevated plasma levels of cholesterol [23]. In the present study, both 100 and 200 mg/kg of ZMME extracts normalized total cholesterol levels. A total of 200 mg/kg of the ZMME extract and clomiphene-treated groups exhibited highly significant activity on the total cholesterol. When comparing the effectiveness of ZMME 200 mg/kg and standard groups, ZMME 200 mg/kg was more potent than the standard clomiphene citrate.

Aerial parts of this plant contain berberine, quercetin, stigmasterol, lanosterol, diosgenin, kaempferol, and sitosterol [24]. Many studies have reported that quercetin and berberine have the potential to treat PCOS. Quercetin suppresses insulin resistance and reduces testosterone and LH levels and is also an effective treatment for PCOS and its complications, including infertility [25,26]. It showed improvement in the inflammatory microenvironment of the ovarian tissue of the PCOS rat model [27]. Another study reported that quercetin has the potential to correct the metabolic and hormonal abnormalities that occur in PCOS [28]. A randomized controlled trial reported that quercetin supplementation decreased resistin plasma levels and gene expression and reduced testosterone and LH concentration in overweight or obese women with PCOS [29]. Oral quercetin supplementation improved the metabolic features of PCOS patients by upregulating the expression of adiponectin receptors and AMPK, thus improving the infertility symptoms of PCOS [30,31]. Many studies have explored anti-androgenic potentials by completely obstructing the PK13 pathway and downregulating the CYP17A1 gene, which controls ovarian steroidogenesis by regulating insulin levels, while some drugs, like metformin, do not affect the CYP17A1 gene. Hence, quercetin is beneficial in normalizing the abnormal levels of progesterone, estrogen, and testosterone, which are known to be present in the ZM plant. The HPTLC fingerprinting of ZM was carried out to identify the chemical components which are responsible for the required pharmacological activity. The HPTLC fingerprint showed the presence of various phytochemicals in the methanol extract, including rutin, quercetin, gallic acid, and ursolic acid. The activity against PCOS might be due to the presence of quercetin in the leaves of the ZM plant.

## 4. Materials and Methods

### 4.1. Collection and Authentication of Plant Material

The leaves of *Ziziphus mauritiana* from the Rhamnaceae family were collected from Nadupalyam pirivu, Coimbatore, India. This was authenticated by Dr. S. Rajan, Survey of medicinal plants and collection unit, Ministry of AYUSH, Government of India.

### 4.2. Preparation and Extraction of Plant Leaves

After cleaning the plant, the stalks, branches, and stems were removed. Only the leaves were taken and dried in the shade; the dried leaves were then chopped and ground using an electronic grinder to obtain a coarse powder. The powdered leaves were extracted with methanol in a Soxhlet extractor. The obtained liquid extract was filtered using Whatman filter paper 1 and evaporated using a Buchi evaporator to obtain the crude plant extract. This was collected and stored safely at −21 °C in the refrigerator until it was used for the study.

### 4.3. Phytochemical Screening

The methanol extract of *Ziziphus mauritiana* (ZMME) was subjected to qualitative chemical analysis such as alkaloids, flavonoids, glycosides, terpenoids, phenols, carbohydrates, proteins, saponins, tannins, and steroids using the methods of Trease, G.E., Khandewal K.R., and Kokate C.K. et al. [32,33,34].

### 4.4. High-Performance Thin Layer Chromatography (HPTLC)

The HPTLC method was developed and validated for the simultaneous estimation of rutin, gallic acid, ursolic acid, and quercetin from the methanolic extract of ZM. HPTLC was performed following the method of Harborne and Wagner et al. [35,36] on silica gel 60 F_254_ 10 cm × 10 cm plates (E. Merck KGaA) with toluene: ethyl acetate: formic acid: methanol (3:6:1.6:0.4) as the mobile phase for the standard and methanolic extract of ZM. The samples (3 μL, 5 μL and 10 μL) were applied to the plate as 6 mm bands. The application of the sample was performed with a CAMAG-Linomat 5 automated spray on a band applicator equipped with a 100 μL Hamilton syringe and was operated with the settings: band length 6 mm, application rate 150 nL/s, application volume 0.2 μL, number of tracks 7, the distance between track 13.3 mm, the position of first track 10 mm and solvent front position 80 mm. The developed plate was sprayed with inert gas and dried at 60 °C in a hot air oven for 5 min. The plate was placed in a photo-documentation chamber (CAMAG TLC scanner 3), and the images were captured under UV light at 254 nm. The R_f_ values and fingerprint data were recorded by WIN CATS software.

### 4.5. Experimental Animals

Adult female Sprague Dawley rats weighing 150–200 g were obtained from Shaqra University and housed in our pharmacy college campus, Al-Dawadmi, and allowed acclimatizing for 14 days. During the study, all animals were maintained in well-ventilated housing conditions of 22 ± 3 °C temperature, 55 ± 5% humidity, and a 12 h light-dark cycle. They were fed with an estrogen-free rodent diet and water ad libitum during the experiment. The study was approved by The Scientific Research Ethics Committee, College of Pharmacy, Male section, Shaqra University, Al-Dawadmi. (Register number ERC SU 20220071).

### 4.6. Acute Toxicity

An acute toxicity study was conducted according to Organization for the Economic Co-operation and Development guidelines 423 (OECD) [37,38]. It is a stepwise procedure with three animals of a single-sex per step. The method used different defined doses (5, 50, 500, 2000 mg/kg body weight), and the substances were ranked and classified according to the Globally Harmonized System (GHS) for classifying the extracts that caused acute toxicity. Three healthy Wister Albino rats weighing 150–200 g were selected for the study. The rats were fasted overnight and provided with water ad libitum. Following a period of fasting, the animals were treated with the methanolic extract ZMME at a dose of 2000 mg/kg body weight orally. As most of the crude extracts posed an LD50 value of more than their 2000 mg/kg body weight, this was used as an initial dose. After oral administration, the rats were observed on an hourly basis for 24 h to access mortality and to detect any changes in the autonomic or behavioral responses viz alertness, aggressiveness, spontaneity, irritability, tremor, corneal reflex, salvation, urination, respiration, convulsion, etc. The rats were observed regularly for 14 days to note mortality or toxic symptoms.

### 4.7. Animal Grouping and Induction of PCOS

Female rats with regular oestrus cyclicity were selected for the experiment. Thirty female Sprague Dawley rats were divided into five groups, and each group contained 6 rats.

Group I (normal control): Received normal saline (0.9% NaCl solution) orally for 21 days.

Group II (PCOS control): Treated with Letrozole (1 mg/kg/day) dissolved in normal saline orally for 21 days.

Group III (ZMME 100 mg/kg): Treated with Letrozole (1 mg/kg/day) dissolved in normal saline orally for 21 days. After PCOS induction, from day 22nd to the 35th (14 days), 100 mg/kg of ZMME was given orally.

Group IV (ZMME 200 mg/kg): Treated with Letrozole (1 mg/kg/day) dissolved in normal saline orally for 21 days. After PCOS induction, from day 22nd to the 35th (14 days), 200 mg/kg of ZMME was given orally.

Group V (Standard control): Treated with Letrozole (1 mg/kg/day) dissolved in normal saline orally for 21 days. After PCOS induction, from day 22nd to 35th (14 days), clomiphene citrates 2 mg/kg was given orally. During the experimental period, the animals were weighed once a week, and a vaginal smear was examined under a microscope to identify the oestrus stage. Only rats with at least three consecutive regular appearances of oestrus stages in order (oestrus cyclicity) were selected for the experiment. If Letrozole (1 mg/kg) treated rats showed irregular ovarian cyclicity, they were considered as PCOS-induced rats.

### 4.8. Oestrus Cycle Determination

Vaginal smears were collected daily at 8:00 a.m., and their oestrus cycles were observed from the beginning of the study to the end of the experimental period. As explained in earlier studies about the stages of the oestrus cycle, nucleated epithelial cells predominate at the proestrus stage, the presence of cornified squamous epithelial cells are at the oestrus stage, a mix of cell types with a predominance of leukocytes and a few nucleated or cornified squamous epithelial cells are in the meta oestrus stage, and predominant leukocytes are present in the dioestrus stage [39].

### 4.9. Biochemical Estimation

#### 4.9.1. Serum Hormonal Analysis

At the end of the experimental period (14 days after the extract treatment), the animals were sacrificed by cervical dislocation, and the blood samples were collected by cardiac puncture. The plasma was separated by centrifugation and stored at −12 °C until they were used for biochemical analysis. After blood collection, the ovaries and uterus were removed and weighed. LH was estimated using ERBA Fertikit LH, Germany. Serum progesterone, estradiol, and testosterone were estimated using the Elba science QuicKey Pro Rat T (testosterone) ELISA kit, QuicKey Pro Rat Pg (progesterone) ELISA kit, and QuicKey Pro Rat E2 (Estradiol) ELISA kit, USA.

#### 4.9.2. Measurement of Total Cholesterol

The total serum cholesterol was measured using the enzymatic kit obtained from ERBA Diagnostics, USA.

#### 4.9.3. Determination of Blood Glucose—Oral Glucose Tolerance Test (OGTT)

On the last day of the study, this test was conducted before and after the induction of PCOS and after treatments with standard and test drugs. Rats were fasted for 12 h and given 2 g/kg of glucose. The blood samples were collected from the tail vein at 0 min (before administration of glucose), 30, 60, and 120 min (after administration of glucose) to detect the blood glycemic level using a Glucometer Accu Chek Active Blood Glucose Meter Kit, India [40].

### 4.10. Histopathological Changes

For the histopathological study, one ovary was removed from one animal of each group and kept in a 10% neutral buffered formalin for 48 h. They were subjected to tissue processing by dehydration using a series of ethanol solutions of increasing concentrations until water-free alcohol was reached. After dehydration, transparent tissue clearing was performed using xylene, which was embedded in paraffin wax into blocks. These blocks were sectioned at 5 µm thickness using a microtome. Then, paraffin-embedded tissue blocks were dewaxed using xylene, rehydrated, and stained with hematoxylin and eosin. The stained slides were examined using a light microscope connected to a camera (Nikon DS-Ri1, India) for capturing images.

### 4.11. Statistical Analysis

These results were given as the mean ± SD of six animals from each group. The statistical analysis was carried out by a one-way analysis of variance followed by Tukey’s multiple comparison tests using Graph pad Prism 5.0 software. *p* values < 0.05 were considered significant.

## 5. Conclusions

The leaves of the *Ziziphus mauritiana* extract showed significant activity for the total cholesterol and different hormones that were studied. Many drugs currently used for the treatment of PCOS have been reported for various adverse effects, and this may lead to failure of the conception. This natural therapy would be beneficial for women who have infertility problems that are related to the improper management of PCOS and failure of pharmacological therapy.

The present research concluded that the leaves of *Ziziphus mauritiana* have potential efficacy in the treatment of PCOS. The beneficial effect of this plant in PCOS might be due to the presence of bioactive compounds such as quercetin, and further clinical investigations are needed to confirm the biological activity of this plant in PCOS.

## Figures and Tables

**Table 1:** HPTLC profile of the *Ziziphus mauritiana* methanolic extract.

R_f_	Height	Area (1/100)	Maximum % Concentration	Assigned Substances
0.05	634.1	141.60	59.54	Rutin
0.68	564.3	113.79	58.27	Quercetin
0.50	518.4	181.44	85.77	Gallic acid
0.78	22.3	85.50	54.96	Ursolic acid
0.00	18.3	23.48	45.04	Unknown
0.02	281.6	74.09	16.02	Unknown
0.19	249.6	125.68	10.22	Unknown
0.13	229.2	55.27	9.38	Unknown
0.63	202.8	56.68	20.94	Unknown
0.74	129.5	27.84	5.30	Unknown
0.31	119.5	55.95	4.89	Unknown
0.72	109.5	13.71	4.48	Unknown
0.38	68.9	18.86	2.82	Unknown
0.10	56.2	11.24	5.28	Unknown
0.81	35.8	8.68	1.47	Unknown
0.54	17.2	5.25	0.71	Unknown
0.34	17.0	3.71	1.75	Unknown
0.85	12.3	1.86	0.50	Unknown

**Table 2:** Effect of drug treatments on body weight and ovarian weight.

Treatment and Dose	Body Weight (g)	Ovary Weight (g)
Normal control	102.6 ± 1.04	0.08 ± 0.0
PCOS control	132.5 ± 2.05 ^##^	0.10 ± 0.0 ^##^
ZMME 100 mg/kg	124.5 ± 2.2 **	0.09 ± 0.0 *
ZMME 200 mg/kg	118.6 ± 2.2 **	0.08 ± 0.0 **
Clomiphene citrate 2 mg/kg	113 ± 1.7 **	0.08 ± 0.0 **

One-way ANOVA followed by Tukey’s multiple comparison test, with values reported as Mean ± SD at *n* = 6, ^##^ *p* ≤ 0.01 vs. normal control and * *p* ≤ 0.05 and ** *p* ≤ 0.01 vs. PCOS control.

**Table 3:** Effect of different treatments on plasma LH, progesterone, estradiol, and testosterone level.

Group	Progesterone (ng/mL)	LH (ng/mL)	Testosterone (ng/mL)	Estradiol (ng/mL)
Normal control	48.83 ± 2.32	07.17 ± 1.47	02.33 ± 1.03	768.67 ± 1.37
PCOS control	17.67 ± 1.03 ^##^	24.33 ± 2.07 ^##^	07.83 ± 1.17 ^##^	127.33 ± 1.63 ^##^
ZMME 100 mg/kg	38.83 ± 2.31 **	17.83 ± 1.17 *	04.83 ± 0.75 **	548.83 ± 1.16 **
ZMME 200 mg/kg	47.83 ± 2.48 **	14.67 ± 1.86 **	04.56 ± 1.47 **	638.66 ± 2.58 **
Clomiphene citrate 2 mg/kg	46.33 ± 1.03 **	10.33 ± 1.03 **	04.16 ± 0.75 **	650.16 ± 1.47 **

One-way ANOVA followed by Tukey’s multiple comparison test with values reported as the Mean ± SD at *n* = 6, ^##^ *p* ≤ 0.01 vs. normal control and * *p* ≤ 0.05 and ** *p* ≤ 0.01 vs. PCOS control.

**Table 4:** Effect of different treatments on total cholesterol.

Group	Total Cholesterol (mg/dL)
Normal control	71.33 ± 1.03
PCOS control	116.33 ± 1.36 ^##^
ZMME 100 mg/kg	81.33 ± 1.03 **
ZMME 200 mg/kg	72.50 ± 0.83 **
Clomiphene citrate 2 mg/kg	74.00 ± 1.41 **

One-way ANOVA followed by Tukey’s multiple comparison test with values reported as the Mean ± SD at *n* = 6, ^##^ *p* ≤ 0.01 vs. normal control and ** *p* ≤ 0.01 vs. PCOS control.

**Table 5:** Effect of different treatments on blood glucose.

Treatment and Dose	Concentration of Blood Glucose (mg/dL)				
	0 min	30 min	60 min	90 min	120 min
Normal control	79 ± 4.5	118.3 ± 7.6	130.2 ± 6.04	157.5 ± 7.5	145.8 ± 5.5
PCOS control	75.8 ± 3.7	120.2 ± 7.3	132.7 ± 7.3	159.3 ± 4.2	149.8 ± 6.9
ZMME 100 mg/kg	79.5 ± 3.7	115.5 ± 13.9	126.7 ± 6.8	162.5 ± 4.2	141.8 ± 6.7
ZMME 200 mg/kg	79.5 ± 7.4	115.2 ± 11.6	126.3 ± 5.04	163.3 ± 6.4	152 ± 7.5
Clomiphene citrate 2 mg/kg	78.5 ± 7.2	115.2 ± 7.05	130.8 ± 5.5	161 ± 7.8	155 ± 6.6

One-way ANOVA followed by Tukey’s multiple comparison test with values reported as the Mean ± SD at *n* = 6.

## Data Availability

All data generated or analyzed during this study are included in this published article.
